# Treatment of immunoglobulin G4-related sialadenitis: outcomes of glucocorticoid therapy combined with steroid-sparing agents

**DOI:** 10.1186/s13075-017-1507-6

**Published:** 2018-01-30

**Authors:** Xia Hong, Yan-Yan Zhang, Wei Li, Yan-Ying Liu, Zhen Wang, Yan Chen, Yan Gao, Zhi-Peng Sun, Xin Peng, Jia-Zeng Su, Zhi-Gang Cai, Lei Zhang, Jing He, Li-Min Ren, Hong-Yu Yang, Zhan-Guo Li, Guang-Yan Yu

**Affiliations:** 1 Department of Oral and Maxillofacial Surgery, Peking University School and Hospital of Stomatology, 22 Zhongguancun South Street, Beijing, 100081 China; 2 Center for Stomatology, Peking University Shenzhen Hospital, Shenzhen, China; 3 Department of Rheumatology and Immunology, Peking University People’s Hospital, 11 Xizhimen South Street, Beijing, 100044 China; 4 Department of Oral Pathology, Peking University School and Hospital of Stomatology, Beijing, China; 5 Department of Oral Radiology, Peking University School and Hospital of Stomatology, Beijing, China

**Keywords:** IgG4-related sialadenitis, Treatment, Glucocorticoid, Steroid-sparing agents, Serum IgG4 level

## Abstract

**Background:**

Immunoglobulin G4-related sialadenitis (IgG4-RS) is a newly recognized immune-mediated systemic disease. Despite its good response to steroid therapy, its treatment protocol is not standardized and the long-term outcome is controversial. The study was conducted to determine the short-term and long-term outcomes of IgG4-RS patients treated with glucocorticoids and steroid-sparing immunosuppressive agents, to analyze secretory function, serological and radiological changes in salivary glands and to assess the usefulness of serum IgG4 level as an indicator of disease activity.

**Methods:**

IgG4-RS patients who were treated for more than 3 months were enrolled. Serological tests, salivary gland function assessment and computed tomography (CT) were performed before treatment and during follow up. The treatment outcomes in the short and the long term were evaluated, and the relationship between serum IgG4 level and salivary gland volume was analyzed.

**Results:**

Glucocorticoids were used in all 43 patients and steroid-sparing immunosuppressive agents in 38 patients (88.4%). The follow-up period was 24.6 ± 14.9 months. Clinical remission was achieved in all patients after induction therapy. During short-term observation, salivary gland secretion significantly increased, and the serum IgG4 levels, the volumes and CT values of submandibular and parotid gland decreased significantly (*P* < 0.001). For long term, relapse occurred in 32.5% patients within 55 months in the regularly treated group, while all seven irregularly treated patients relapsed. However, the relapse-free survival curves were not significantly different between the steroid monotherapy and the combination therapy groups (*P* = 0.566). Submandibular glands, lacrimal glands, sublingual glands, nasal and paranasal cavity were commonly relapsing organs. In clinically stable patients, a serologically unstable condition occurred in 54.9% patients within 55 months and medication adjustment was performed accordingly. Volume changes in the submandibular and parotid glands were associated with serum IgG4 levels and time of follow up (*R*^2^_adjusted_ = 0.905, *P* < 0.0001 and *R*^2^_adjusted_ = 0.9334, *P* < 0.0001, respectively).

**Conclusions:**

The combination of glucocorticoid and steroid-sparing agents could be effective for treating IgG4-RS, and restoring salivary gland function. Serum IgG4 levels could predict disease activity.

**Electronic supplementary material:**

The online version of this article (doi:10.1186/s13075-017-1507-6) contains supplementary material, which is available to authorized users.

## Background

Immunoglobulin G4-related disease (IgG4-RD) is an immune-mediated condition characterized by dense lymphoplasmacytic infiltrates, storiform fibrosis, frequent elevations of serum IgG4 and good response to steroid therapy [1]. Almost every organ in the body could be affected in this systemic disease, and salivary glands are among the most commonly involved sites, a condition known as immunoglobulin G4-related sialadenitis (IgG4-RS) [2–4].

Glucocorticoids are regarded as the first-line agents for inducing remission of IgG4-RS. However, the optimal therapeutic strategy has not yet been established [5]. Relapses are common both during and after treatment, and therefore, maintenance treatment seems essential [6]. Steroid-sparing agents, such as azathioprine, mycophenolate mofetil and cyclophosphamide, have also been used to treat IgG4-RD, but the optimal timing of their administration is controversial, and their outcomes are unclear [5, 7]. Xerostomia is commonly reported in IgG4-RS [8], but the long-term outcomes of the secretory function have not yet been studied. The diagnostic value of serum IgG4 level is controversial, and its role in predicting disease activity is unclear [9].

To resolve the aforementioned issues, we conducted a study to determine the short-term and long-term outcomes of patients with IgG4-RS treated with glucocorticoids combined with steroid-sparing immunosuppressive agents. We analyzed secretory function and the serological and radiological changes, and evaluated the usefulness of serum IgG4 level as an indicator of disease activity.

## Methods

The study protocol was approved by the Ethics Committee for Human Experiments of Peking University School of Stomatology. Informed consent was obtained from all the patients included in this study.

### Patients

The study included all patients with confirmed IgG4-RS who were diagnosed, treated and followed up for more than 3 months at the Department of Oral and Maxillofacial Surgery, Peking University School of Stomatology between August 2011 and April 2017. IgG4-RS was diagnosed based on the comprehensive diagnostic criteria for IgG4-RD [5]: (a) persistent (>3 months) swelling of single or multiple major salivary glands; (b) serum IgG4 concentration >1350 mg/L; (c) histopathological and immunohistochemical examinations of submandibular gland biopsy specimens showing marked lymphocyte and plasmacyte infiltration, fibrosis and infiltration of IgG4+ plasma cells with IgG4+/IgG+ cells >40% and IgG4+ plasma cells >10/high-power field; and (d) exclusion of other diseases that present with glandular swellings, such as sarcoidosis and lymphoproliferative disease. Patients who suffered from contraindications to glucocorticoid treatment or had history of glucocorticoid treatment were excluded from the study. The patients’ basic information and medical history were recorded. Serological tests, salivary gland function assessment and computed tomography (CT) were performed before treatment and during follow up.

### Serological tests

Serum IgG4 levels were measured using the Array 360 Immunoassay Assay Protein Serology Chemistry Analyzer system (Beckman Coulter, Fullerton, CA, USA).

### Salivary gland function assessments

The degree of subjective oral dryness was assessed using the summated xerostomia inventory (SXI) [8, 10]. Saliva flow rates were calculated by collecting the whole saliva samples at rest and under stimulation with 2.5% citric acid solution for 5 minutes each [11]. Scintigraphy with ^99m^Tc-pertechnetate was performed according to a standardized protocol [12]. Both the parotid and submandibular glands were evaluated and the secretion index (SI) was calculated (Additional file 1).

### CT assessments

CT scanning was performed using an eight-slice scanner (BrightSpeed; GE Medical Systems, Waukesha, WI, USA). The CT data in digital imaging and communications in medicine (DICOM) format were imported to iPlan CMF (BrainLAB, AG, Germany). The margins of the right and left parotid and submandibular glands were marked with this software, and then reconstructed using volume rendering [13]. The images were reconstructed three times, and the mean gland volume and mean CT value of the bilateral glands were recorded.

### Treatment regimen

The preferred treatment regimen was intravenous methylprednisolone at a dose of 200 mg/day for 3 days and 40 mg/day for another 3 days, followed by 0.6 mg/kg/day oral prednisone for 2 weeks, tapered by 5 mg every 2 weeks until a daily dose of 30 mg was reached and then tapered by 2.5 mg every 2 weeks to reach a maintenance dose of 5 mg/day. In some patients, only oral prednisone was administered at the initial dose of 0.6 mg/kg/day for 4 weeks before the gradual tapering. The preferred initial immunosuppressive agent for combination therapy was cyclophosphamide at a dose of 400 mg once every 2 to 4 weeks; other immunosuppressive agents, including azathioprine and leflunomide, could also be used at doses of 2 mg/kg/day and 10 mg/day, respectively. Mycophenolate mofetil at 250 mg three times a day (TID) was used as the initial immunosuppressive agent in children.

### Treatment response assessments

Treatment response was assessed every 3 to 6 months using a comprehensive evaluation strategy. The short-term outcomes were collected at 3 months and the long-term outcomes were collected in patients treated for more than 12 months. The following parameters were evaluated:

- Sizes of the salivary glands: physical examinations of the major salivary glands were performed at each follow up as the basic evaluation, and CT assessment of the volumes and mean CT values of the parotid and submandibular glands were performed at 3 months for the first time and then once every 6 to 12 months, or when relapse was suspected during follow up. In comparison to the pretreatment volumes, reductions ≥30%, ≥15% but <30%, and <15% in the short term indicated complete, partial, and poor remission, respectively. Increases ≥30%, ≥15% but <30%, and <15% compared to the short-term value, or the value at 6 months if data from the 3-month visit were absent, indicated complete, partial, and minor relapse of the salivary glands, respectively.
- Function of salivary glands: summated xerostomia inventory (SXI) scores were recorded as the subjective evaluation of xerostomia symptoms. Saliva flow rate was analyzed as the secretion index (SI) for objective evaluation.
- Serological examinations: serum IgG4 levels were tested at each follow up. A sustained increase >20% during follow up as compared with the short-term level was considered to be evident increase.
- Extra-salivary involvement assessment: symptoms such as nasal obstruction or allergic rhinitis, audition decrease, and abdominal pain were noted. Physical and imaging examination of the lacrimal gland, nasal cavity, lymph nodes, and pancreas, etc. was performed.

Clinical remission was defined as complete or partial remission on CT, and improvement in extra-salivary involvement. Clinical relapse was defined by re-enlargement of salivary glands or new appearance or reappearance of extra-salivary involvement after clinical remission. Patients with obvious aggravation of subjective symptoms such as xerostomia or evident increase in serum IgG4 during follow up were suspected to be relapsing and in need of further evaluation. However, isolated increase in serum IgG4 did not indicate clinical relapse, but was defined as a serologically unstable condition.

### Statistical analysis

Statistical analyses were performed using IBM SPSS Statistics 22 (IBM, Armonk, NY, USA) and Statistical Analysis System software 9.3 (SAS Institute, Cary, NC, USA). Kolmogorov-Smirnov tests were performed before parametric tests and serum IgG4 concentrations in mg/L were log-transformed to approximate normality. Statistical differences were analyzed using the Student *t* test and paired *t* test, Mann–Whitney U test and chi-square test. Multiple linear regression analysis was used to analyze the association between serum IgG4 and parotid and submandibular gland volumes, and Kaplan–Meier analysis was used to evaluate the relapse-free survival rate. *P* < 0.05 was considered to indicate significant differences.

## Results

### General information

Forty-three patients were included in this study. Their baseline information is listed in Table 1. There were 38 patients (88.4%) treated with glucocorticoid combined with steroid-sparing agents. Among them, treatment was initiated with intravenous methylprednisolone in 35 patients and with oral prednisone in 3 patients. Steroid-sparing agents including cyclophosphamide, leflunomide, azathioprine and mycophenolate mofetil were administered to 32, 4, 1 and 1 patient, respectively. The other five patients were treated with steroid monotherapy, including four patients starting treatment with intravenous methylprednisolone and one with oral prednisone. The mean follow-up period was 24.6 ± 14.9 months.

**Table 1:** Baseline information on patients with IgG4-RS

Characteristic		Value
Age (years)		51.8 ± 14.4
Male:female ratio		0.54:1
Enlargement of major salivary glands		
SMG	Bilateral, *n* (%)	42 (97.7)
	Unilateral, *n* (%)	1 (2.3)
PG	Bilateral, *n* (%)	30 (69.8)
	Unilateral, *n* (%)	3 (7.0)
Sublingual gland, *n* (%)		30 (69.7)
Accessory parotid gland, *n* (%)		7 (16.3)
Extra-salivary involvement, *n* (%)		39 (90.7)
Enlargement of lacrimal gland, *n* (%)		31 (72.1)
Enlargement of cervical lymph nodes, *n* (%)		33 (76.7)
Rhinosinusitis, *n* (%)		22 (51.2)
Asthma, *n* (%)		2 (4.7)
Autoimmune pancreatitis, *n* (%)		2 (4.7)
Interstitial pneumonia, *n* (%)		2 (4.7)
Sclerosing cholangitis, *n* (%)		1 (2.3)
Elevated serum IgG4 level, *n* (%)		42 (97.7)

*IgG4-RS* IgG4-related sialadenitis, *SMG* submandibular gland, *PG* parotid gland

### Short-term outcomes

In the very short term, the decrease in gland size was observed from the second day of treatment in all patients who received intravenous methylprednisolone. After the 6-day course of full-dose intravenous steroid therapy, physical examination showed that all the involved salivary glands regressed as did the enlarged lacrimal glands and lymph nodes, and the SXI decreased from 8.34 ± 2.61 to 5.97 ± 1.12 (*P* < 0.01). In the four patients started on oral prednisone, the reduction in gland size was observed 2–4 weeks after treatment, and the symptoms gradually improved.

Thirty-five patients returned for their first post-treatment assessment at 3 months. Although enlargement of the submandibular and parotid glands was still observed on physical examination in two patients (5.7%) and seven patients (20.0%), respectively, there was a marked decrease in size in all patients. The sizes of the involved sublingual glands and accessory parotid glands were also reduced. On measurement of glandular volume on CT, the volumes and CT values of the submandibular and parotid glands decreased significantly (Table 2, Additional file 2). The mean reduction rate in the size of the submandibular gland was 41.4 ± 14.2%. Complete remission in the submandibular gland occurred in 24 patients (80.0%), and partial remission occurred in 6 patients (20.0%). In contrast, the mean reduction rate in the size of the parotid glands was 7.2 ± 12.2%, and remission in the parotid gland was observed in six patients (18.8%), including three patients with complete remission and three with partial remission. The baseline CT values for the parotid glands with complete or partial remission were significantly higher than the parotid glands with poor remission (29.66 ± 11.76 Hounsfield units (HU) vs –6.01 ± 12.43 HU, *P* < 0.001).

**Table 2:** Comparison of different parameters before treatment and at 3 months after treatment in patients with IgG4-RS

	Number	Pretreatment	3 Months after treatment	*P* value
Clinical examination				
Enlargement of SMG, *n* (%)	35	35 (100.0)	2 (5.7)	<0.001
Enlargement of PG, *n* (%)	35	27 (77.1)	7 (20.0)	<0.001
Salivary gland secretory function				
SXI	35	8.46 ± 2.44	6.14 ± 1.19	<0.001
Whole saliva flow rate at rest (g/5 min)	35	0.84 ± 0.56	1.40 ± 0.76	<0.001
Whole saliva flow rate after acid stimulation (g/5 min)	35	8.81 ± 3.65	10.96 ± 4.05	<0.001
SI of PG (%)	23	51.10 ± 9.29	53.11 ± 9.04	0.321
SI of SMG (%)	20	27.88 ± 8.58	37.58 ± 10.42	<0.001
Log_2_ Serum IgG4 level (mg/L)	35	13.05 ± 1.19	11.06 ± 1.11	<0.001
CT scan				
Volume of SMG (cm^3^)	30	13.54 ± 3.84	7.78 ± 2.69	<0.001
CT value of SMG (HU)	30	37.83 ± 8.45	31.52 ± 5.87	0.001
Volume of PG (cm^3^)	32	33.45 ± 10.06	30.56 ± 8.54	0.004
CT value of PG (HU)	32	0.65 ± 18.66	-12.01 ± 13.09	<0.001

*IgG4-RS* IgG4-related sialadenitis, *SXI* summated xerostomia inventory, *SI* secretion index, *SMG* submandibular gland, *PG* parotid gland, *CT* computed tomography, *HU* Hounsfield units

Secretory function assessment showed that the symptoms of dry mouth were relieved according to the SXI scores, and objectively, the whole saliva flow rate at rest and after acid stimulation also increased significantly (Table 2). Scintigraphy showed large increases in the SI in the submandibular glands.

Serological tests showed that the serum IgG4 levels significantly decreased (Table 2), and the mean difference in the log-transformed value was 1.99 ± 0.77. Patients with higher baseline IgG4 had a more rapid drop in IgG4 after treatment, but were less likely to achieve low or normal levels (Additional file 3).

The medical examination and imaging showed remission of all extra-salivary involvement. Considering the clinical and radiological outcomes together, initial clinical remission was achieved in all patients.

### Long-term outcomes

#### Clinically relapsing cases

Thirty-five patients were followed for more than 12 months. All but seven patients followed the treatment regimens strictly. In general, clinical relapse was observed in 13 patients in the long term. The common locations of recurrence were the submandibular gland, lacrimal gland, sublingual gland and the nasal and paranasal cavity (Table 3), and the serum IgG4 level was obviously increased in all but one relapsing patient.

**Table 3:** Organ recurrence in relapsing patients with IgG4-RS

Involved organs	Relapsing organs/pre-treatment involved organs, *n*/*n* (%)
Submandibular glands	13/13 (100.0)^a^
Parotid glands	4/12 (33.3)
Sublingual glands	8/11 (77.8)
Accessory parotid glands	2/2 (100.0)
Lacrimal glands	13/13 (100.0)
Cervical lymph nodes	6/11 (55.6)
Nasal and paranasal cavity	8/8 (100.0)
Pancreas	0/1 (0.0)
Bile duct	0/1 (0.0)

^a^Patients with relapse in the submandibular glands included 11 with complete relapse and 2 with partial relapse identified by computed tomography

*IgG4-RS* IgG4-related sialadenitis

**Fig. 1:**
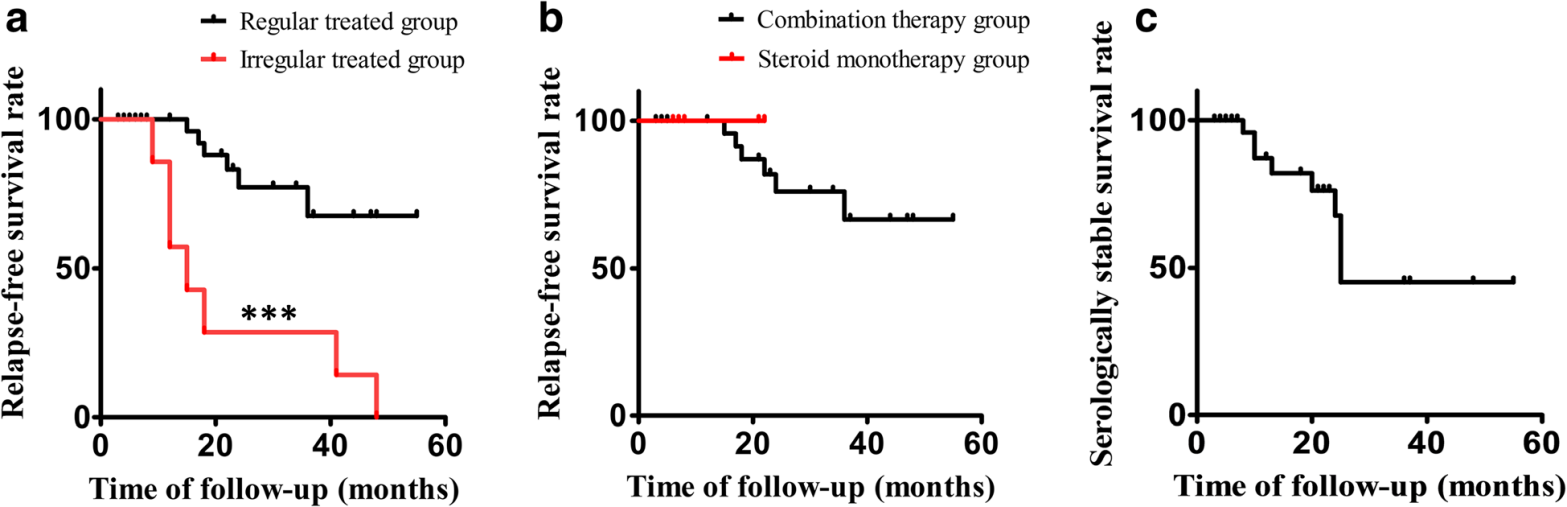
Kaplan–Meier analysis of treatment outcomes in patients with immunoglobulin G4 (IgG4)-related sialadenitis. **a** Partial or complete relapse occurred in 32.5% of patients within 55 months in the regularly treated group, while in the irregularly treated group, all the patients relapsed. **b** The relapse-free survival curves for the groups of patients treated with or without steroid-sparing agents are not significantly different (*P* = 0.566). **c** The serologically stable survival curve shows that a serologically unstable condition occurred in 54.9% patients within 55 months in regularly treated patients

Among the regularly treated patients, clinical relapse occurred in six patients, at 15 to 36 months (median: 20 months) after the start of medication. The baseline features were not significantly different between clinically relapsing and clinically stable groups (Additional file 4: Table S1). Kaplan–Meier analysis showed that relapse occurred in 32.5% patients (standard error: 0.121) within 55 months (Fig. 1a). All the clinically relapsing patients were treated with glucocorticoid combined with steroid-sparing agents. The relapse-free survival curves were not significantly different between the steroid monotherapy and the combination therapy groups (*P* = 0.566, Fig. 1b). The annual relapse rate was 8.80% in all the regularly treated patients and was 9.55% in the combination therapy group.

The other seven patients were not treated regularly; steroids were either tapered faster than advised (n = 1) or the patients discontinued medication themselves (n = 6). In these patients, the relapse was noticed 3 to 24 months (median: 6 months) after the discontinuation of treatment. The relapse rate was 100% within 48 months, which was significantly higher than regularly treated patients (*P* < 0.001, Fig. 1a).

Relapses were treated with oral prednisone at a dose of 0.4–0.6 mg/kg/day combined with immunosuppressive agents, including cyclophosphamide (n = 1), azathioprine (n = 2), mycophenolate mofetil (n = 2) and leflunomide (n = 2). Two patients refused immunosuppressive agents, and steroid monotherapy was adopted. All the glands involved in disease relapse gradually reduced in size, and the patients were followed for another 3 to 26 months without a second clinical relapse. The other four relapsing patients were lost to follow up.

#### Serologically unstable cases

Twenty-two patients were followed for more than 12 months without clinical relapse. An evident increase in serum IgG4, or the serologically unstable condition, was observed in seven of these patients. According to the Kaplan–Meier analysis, the serologically unstable condition occurred in 54.9% patients (standard error: 0.152) within 55 months in regular treated patients, and the median timing of serum IgG4 elevation was 25.0 months (Fig. 1c). The differences in baseline features between the serologically stable and serologically unstable groups were not significant (Additional file 4: Table S2).

Combined with the elevation in serum IgG4, minor relapse in the submandibular glands was observed. The dose of prednisone or immunosuppressive agents was adjusted accordingly in three patients and observation without dose adjustment was adopted in the other four patients. However, a further increase in serum IgG4 and the size of the submandibular glands on CT were observed. To prevent clinical relapse, dose adjustment was adopted later.

#### Maintenance therapy

For maintenance therapy, the mean dose of prednisolone was 5.19 ± 1.83 mg/day in clinically stable patients, which was lower than the maintenance dose of 7.50 ± 2.50 mg/day for clinically relapsing patients (*P* = 0.015). Except for four patients who were within the tapering stage after dose adjustment, 74.1% patients were treated with no more than 5 mg/day prednisolone, but only one patient was allowed to discontinue glucocorticoid. The average maintenance dose for serologically stable and serologically unstable patients was not significantly different (4.75 ± 1.51 mg/day vs 6.50 ± 2.24 mg/day, *P* = 0.061).

The use of steroid-sparing agents was stopped in 15.4% patients after 31 to 36 months of administration. Among the patients started on cyclophosphamide, the medicine was changed to other steroid-sparing agents, including lefunomide, azathioprine and mycophenolate mofetil, in 42.9% patients after 9 to 50 months (median: 25 months).

### Relationship between serum IgG4 level and salivary gland volume

**Fig. 2:**
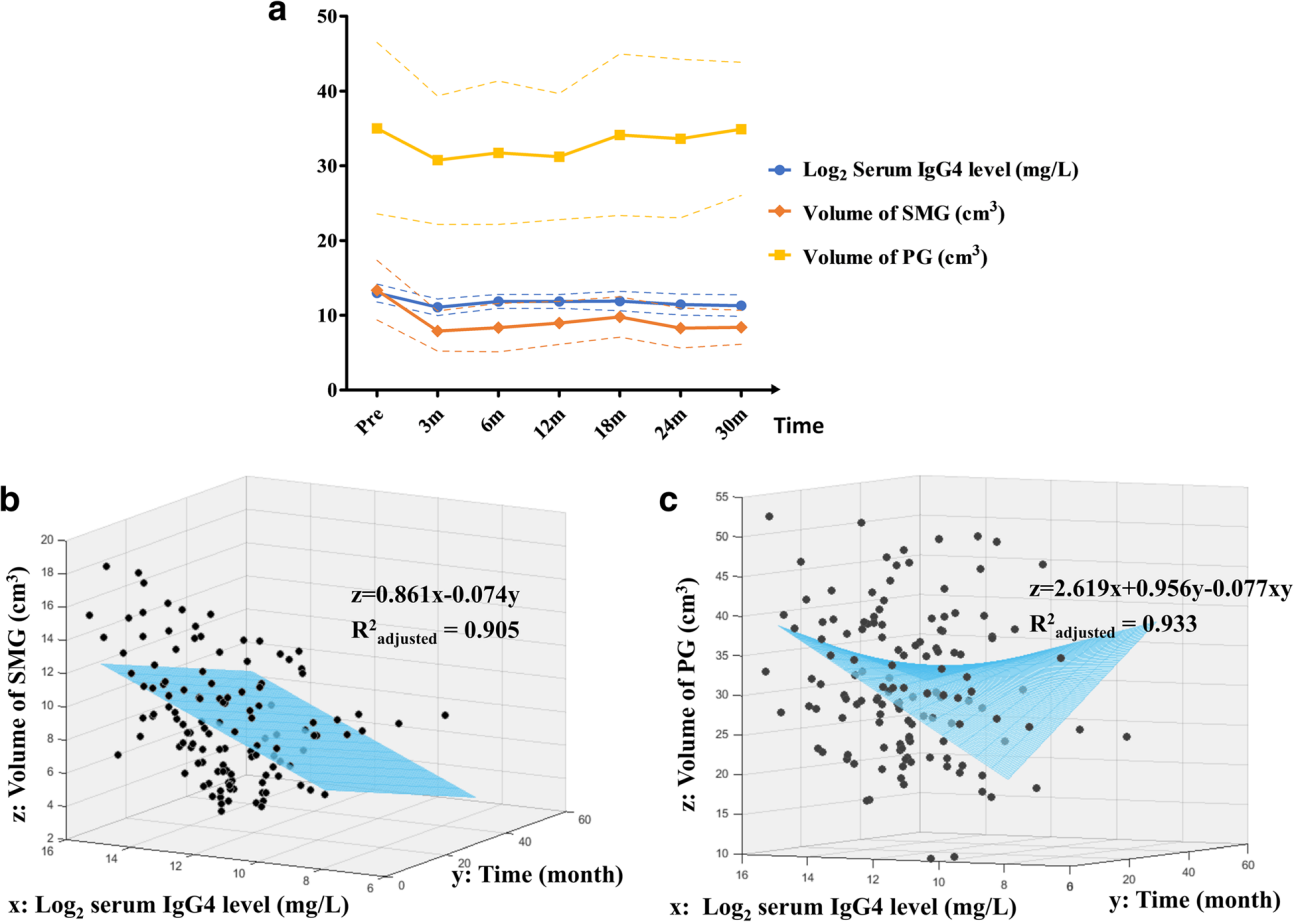
Association between serum immunoglobulin G4 (IgG4) levels and volumes of the submandibular gland and parotid gland. **a** Mean volume (S.D. in dotted curves) of the submandibular gland (SMG), parotid gland (PG) and log_2_ serum IgG4 at each follow up time point exhibit similar variation. Three-dimensional scatter plot and regression function shows the correlation between SMG volume and log_2_ serum IgG4 and the parallel time of follow up (*P* < 0.0001 (**b**)), and correlation between PG volume and log_2_ serum IgG4, time of follow up, and their interaction term (*P* < 0.0001 (**c**))

The mean volumes of the submandibular glands and parotid glands, and the log_2_ serum IgG4 levels at different time points during follow up showed that they varied similarly over time (Fig. 2a). To further identify their relationships, multiple linear regression was performed, which showed that the volume of submandibular gland was positively correlated with log_2_ serum IgG4 level and was negatively correlated with the parallel time of follow up (*R*^2^_adjusted_ = 0.905, *P* < 0.0001, Table 4, Fig. 2b). The volume of the parotid gland was positively correlated with log_2_ serum IgG4 level and the parallel time of follow up, and was negatively correlated with their interaction term (*R*^2^_adjusted_ = 0.9334, *P* < 0.0001, Table 4, Fig. 2c). These suggested that the alterations in serum IgG4 levels might reflect changes in submandibular and parotid gland volumes, and when the serum parameter was kept stable, the volumes would still change slightly over time after treatment.

**Table 4:** Regression models for salivary gland volumes in patients with IgG4-RS

	β	Standard error	*t*	*P* value
Regression model for volume of SMG				
Log_2_ serum IgG4 (mg/L)	0.861	0.029	29.97	<0.0001
Time of follow up (months)	-0.074	0.023	-3.21	0.0017
Regression model for volume of PG				
Log_2_ serum IgG4 (mg/L)	2.619	0.076	34.27	<0.0001
Time of follow up (months)	0.956	0.385	2.48	0.0143
Interaction term	-0.077	0.033	-2.30	0.0229

*IgG4-RS* IgG4-related sialadenitis, *SMG* submandibular gland, *PG* parotid gland

### Side effects

Myelosuppression was observed in one patient using azathioprine, and gastrointestinal reaction was reported in another patient using cyclophosphamide. Mild liver function damage, acne and temporal hyperglycemia occurred in one, two and three patients, respectively, which were regarded as steroid-related. Other side effects were not observed during follow up.

## Discussion

For the treatment of IgG4-RD, a recent systematic review showed that “no therapies” were carried out in 36.6% of patients with IgG4-RS [7]. Despite the possibility of spontaneous remission, long-term data on the “wait and see” strategy are absent. In our series, spontaneous or temporary remission had not been reported by any patient. Our previous study revealed a lower secretory capacity in patients with higher grades of histopathological fibrosis and inflammation [11]. Negative correlation between the increase in salivary flow rate after treatment and the disease duration before treatment has also been reported [14]. It seems that most of the patients with IgG4-RS could not benefit from the “wait and see” strategy in aspect of salivary gland function preservation. On the other hand, remarkable improvement was noted in saliva secretion according to both subjective and objective parameters in our study, and the condition remained stable during the tapering and maintenance periods. Despite there being no statistical difference among patients with different histopathological stage of disease (data not shown), which may be related to the small sample size, we believe that the more severe the fibrosis, the less likely it is that functional glandular tissue exists. Thus, to ensure better recovery of salivary gland function and to minimize the impairment to the whole body, we suggest that treatment be started as soon as the diagnosis is definite.

It is widely accepted that glucocorticoids are the first-line agents for remission induction, and initial remission could be achieved in the majority of patients [15]. Despite the recommendation of an initial dose of oral prednisone of 30–40 mg/day for 4 weeks, there is no controlled study on the optimal treatment regimen, including the starting dose, the tapering protocol and the timing of glucocorticoid discontinuation [7, 15]. Although intravenous steroid treatment has seldom been reported in IgG4-RD [16–18], we prefer a full-dose intravenous steroid therapy protocol in all patients without high risk of side effects. With this regimen, all symptoms and signs of IgG4-RS obviously improved after the second day of treatment. In addition, the dose in our regimen was lower than the traditional steroid pulse therapy, and this may reduce the dose-dependent toxic effect on hepatocytes and other side effects. According to our experience in the treatment of other autoimmune diseases, steroid pulse therapy may allow more rapid tapering of oral glucocorticoids and lower daily maintenance dose. This has long-term benefits, which are probably attributable to effects on both genomic and non-genomic pathways during the intravenous pulse therapy [19, 20]. In our cohort, only four patients were treated without intravenous pulse therapy and the follow-up period was not sufficiently long. As a result, the long-term outcome may not be comparable. A well-designed clinical trial is needed to further evaluate the effectiveness of the full-dose intravenous steroid therapy versus the traditional oral prednisone treatment strategy in IgG4-RD.

The application of steroid-sparing immunosuppressive agents in IgG4-RS is controversial. Some specialists disagree with the addition of immunosuppressive agents at the beginning, while other experts think it is acceptable in some patients [15]. Several studies have reported that steroid-sparing agents seem unable to significantly reduce the relapse rate of IgG4-RD [21], while Fei et al. and Gupta et al. reported favorable responses in patients with IgG4-RD treated with glucocorticoids and steroid-sparing immunosuppressive agents [22, 23]. In our series, no difference was observed between the groups treated with or without steroid-sparing agents. However, the size of the group treated with steroid alone was rather small and the duration of follow up was not sufficiently long. The annual relapse rate was 8.35% in regularly treated patients, lower than the rate of 11.5% reported by Yamamoto et al. [6]. However, our low annual relapse rate could also be related to the dose adjustments made in response to increased serum IgG4 levels. Additionally, the use of immunosuppressive agents helped to minimize the maintenance dose of prednisone, which helps reduce the long-term toxicity of glucocorticoids [19]. Yamamoto et al. reported that only 52.8% patients were treated with < 5 mg/day prednisolone for the maintenance of clinical remission [6], while in our study, the percentage was 74.1%. Cyclophosphamide was chosen as the preferred immunosuppressive agent as it is a classic and widely used immunosuppressive agent that is mainly related to B cell activation [24]. A recent randomized controlled trial (RCT) on IgG4-RD also showed that glucocorticoids combined with cyclophosphamide treatment had a better effect and lower relapse rate than steroid monotherapy during the observation period of 12 months [22]. The long-term toxicity of cyclophosphamide had not been observed in our study. However, further clinical trials and longer-term follow up are necessary, and the efficacy of different immunosuppressive agents needs to be compared. It has been suggested that B cell depletion, such as rituximab, could be effective in many refractory conditions, and XmAb5871, a specific plasmablast-targeted monoclonal antibody, is currently in phase II development for IgG4-RD treatment [25–27]. Targeted medications might be used for the treatment of IgG4-RD in the future. However, as all the patients in our series had acceptable outcomes without severe side effects, and because of financial considerations, rituximab has not yet been recommended for our patients.

In our study, six patients stopped medication by themselves, and relapse was observed in all of them, indicating the high risk of relapse after glucocorticoid discontinuation. Similar results were also reported in autoimmune pancreatitis and IgG4-related cholangitis [28, 29]. Thus, the therapeutic course of IgG4-RS is controversial, and the discontinuation of glucocorticoids is questionable. Several clinicians recommend maintenance therapy for up to 3 years. However, the relapse rate still seems to be high [6, 15]. Furthermore, whether or not immunosuppressive agents or monoclonal antibodies can replace glucocorticoids remains unknown.

Because of the lack of an established standard for the evaluation of therapeutic outcomes, the responses to treatment in different studies may not be comparable [7]. Although an IgG4-RD responder index has been developed for systemic evaluation and has good consistency with physician global assessment [30], the specific standard for assessing each single organ remains unclear. Mikulicz’s Disease Assessment Questionnaire has been used for outcome evaluation, but it is based on subjective judgment [6]. In this study, we came up with a criterion mainly based on the variation in salivary gland volume on CT, which is quantitative, and much more sensitive and objective than physical examination. A similar method has been used in the evaluation of the outcomes in the lacrimal glands in IgG4-RD, though it did not involve CT volume rendering [31]. We set 15% and 30% as the cutoff values for partial and complete remission/relapse, respectively, for both the parotid and submandibular glands, and found good consistency with clinical observations in the submandibular glands and in parotid glands with high CT values. Therefore, we believe that therapeutic evaluation should be systematic and organ specific.

Though being an important feature of IgG4-RD, the significance of serum IgG4 concentration is ambiguous. On one hand, serum IgG4 is an item for consideration in the diagnostic criteria for IgG4-RD; on the other hand, several studies have demonstrated its poor diagnostic utility [9, 32]. Higher baseline serum IgG4 could be related to wider systemic involvement, greater risk of relapse and shorter time to relapse [4, 33, 34], indicating its role in revealing the general condition of the body and predicting the prognosis of the disease. In this study, multiple linear regression revealed strong association between serum IgG4 and parotid and submandibular gland volume, suggesting the significance of serum IgG4 concentrations in reflecting volumes in the salivary glands, and subsequently the disease activity. The variation in salivary gland size could be slight or undetectable by physical examination or traditional radiological imaging, but the serological parameter seems to be more sensitive. In the four patients with obvious serum IgG4 elevation, a further increase in serum IgG4 and salivary glands volumes on CT occurred during follow up. Based on these results, we suggest that serum IgG4 concentration may be useful in predicting the activity of the disease, and dose adjustments could be considered when serum IgG4 increases significantly, even if clinical relapse is absent.

## Conclusions

The combination of glucocorticoids and steroid-sparing agents is effective in treating IgG4-RS. A full-dose intravenous steroid therapy protocol could be beneficial for induction, and maintenance therapy seems necessary. The secretory function of the salivary glands recovers after treatment and remains stable in clinically stable cases. Serum IgG4 level is associated with the activity of IgG4-RS, and reasonable regulation of dosage could be considered when there is obvious elevation in serum.

## Additional files

Additional file 1: Detailed information on scintigraphy assessments. (PDF 194 kb)

Additional file 2: Supplementary figure for volume rendering of submandibular and parotid glands before and after treatment. (PDF 285 kb)

Additional file 3: Supplementary figure for relationship between the short-term IgG4 levels after treatment and the baseline levels. (PDF 289 kb)

Additional file 4: Supplementary tables for comparing the baseline features between different groups. (PDF 298 kb)

## Abbreviations

CT: Computed tomography
HU: Hounsfield units
IgG4-RD: Immunoglobulin G4-related disease
IgG4-RS: Immunoglobulin G4-related sialadenitis
PG: Parotid gland
SI: Secretion index
SMG: Submandibular gland
SXI: Summated xerostomia inventory
