# Supplementary material for: Treatment of immunoglobulin G4-related sialadenitis: outcomes of glucocorticoid therapy combined with steroid-sparing agents
Source: Arthritis Res Ther. 2018 Jan 30;20:12. doi: 10.1186/s13075-017-1507-6 (PMC5791187; doi:10.1186/s13075-017-1507-6)
Supplement: Supplementary file 1 — Detailed information on scintigraphy assessments. (PDF 194 kb) [file 13075_2017_1507_MOESM1_ESM.pdf]

Scintigraphy assessments

Scintigraphy with ^{99m}Tc-pertechnetate was performed according to a standardized protocol [1]. The “secretion phase” was defined as the period starting from stimulation with 2.5% citric acid solution until the minimum value reached within 30 min after stimulation. The secretion index (SI) was calculated using the following formula:

SI = (maximum value before stimulation with citric acid – minimum value after stimulation with citric acid)/(maximum value before stimulation with citric acid – background) × 100%

Both the parotid and submandibular glands were evaluated, and the mean value of the right and left sides was taken to be the SI of each type of salivary gland.

References

1. Aung W, Yamada I, Umehara I, Ohbayashi N, Yoshino N, Shibuya H. Sjögren's syndrome: comparison of assessments with quantitative salivary gland scintigraphy and contrast sialography. J Nucl Med. 2000;41:257–62.
