# Supplementary material for: Treatment of immunoglobulin G4-related sialadenitis: outcomes of glucocorticoid therapy combined with steroid-sparing agents
Source: Arthritis Res Ther. 2018 Jan 30;20:12. doi: 10.1186/s13075-017-1507-6 (PMC5791187; doi:10.1186/s13075-017-1507-6)
Supplement: Supplementary file 2 — Supplementary figure for volume rendering of submandibular and parotid glands before and after treatment. (PDF 285 kb) [file 13075_2017_1507_MOESM2_ESM.pdf]

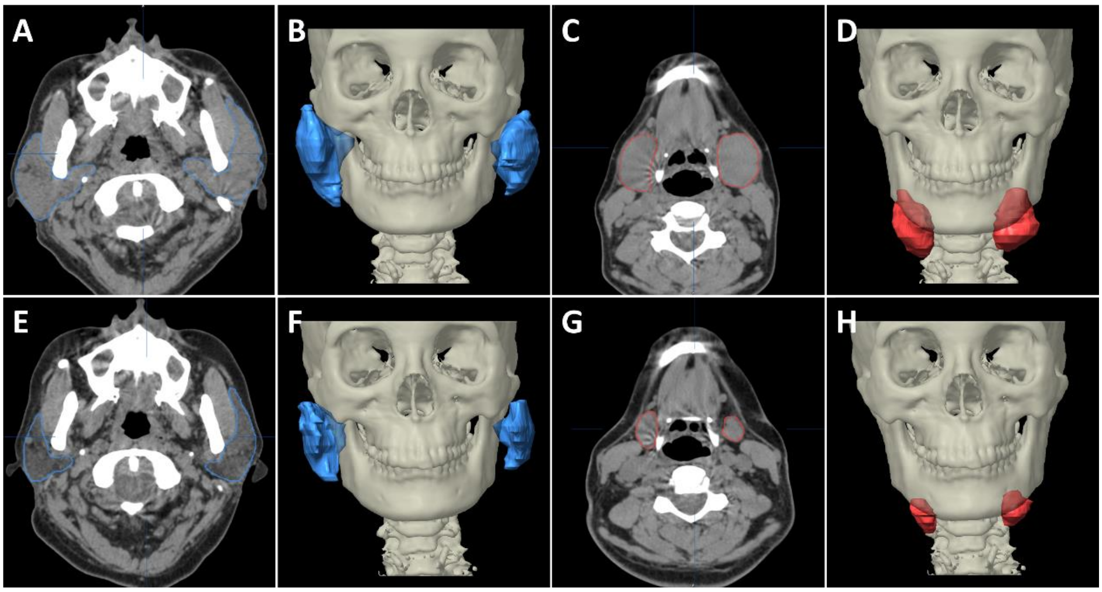

Supple Fig. 1 Decrease of volumes and CT values of submandibular and parotid glands in IgG4-related sialadenitis patients.

The margins of parotid and submandibular glands are marked on axial CT scan and then reconstructed using volume rendering. The volumes and CT values of parotid glands (**A, B**) and submandibular glands (**C, D**) are higher than normal before treatment, and decrease obviously at 3 months (**E, F, G, H**). With the help of volume rendering, the alternations are quantifiable.
