# Supplementary material for: Treatment of immunoglobulin G4-related sialadenitis: outcomes of glucocorticoid therapy combined with steroid-sparing agents
Source: Arthritis Res Ther. 2018 Jan 30;20:12. doi: 10.1186/s13075-017-1507-6 (PMC5791187; doi:10.1186/s13075-017-1507-6)
Supplement: Supplementary file 3 — Supplementary figure for relationship between the short-term IgG4 levels after treatment and the baseline levels. (PDF 289 kb) [file 13075_2017_1507_MOESM3_ESM.pdf]

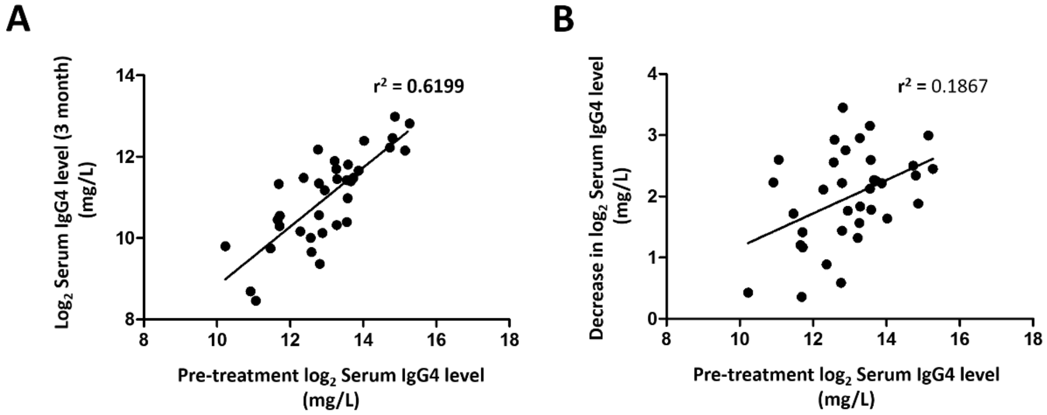

Supple Fig. 2 Changes of serum IgG4 levels in short-term after treatment in IgG4-related sialadenitis patients.

Both the Log_2 serum IgG4 level at 3 months and the decrease value were positively correlated with pre-treatment Log_2 Serum IgG4 level (**A**, $P < 0.0001$ and **B**, $P = 0.0095$, respectively), indicating that patients with higher baseline IgG4 levels showed a greater decrease after treatment, but were less likely to achieve lower or normal levels.
