# Supplementary material for: Treatment of immunoglobulin G4-related sialadenitis: outcomes of glucocorticoid therapy combined with steroid-sparing agents
Source: Arthritis Res Ther. 2018 Jan 30;20:12. doi: 10.1186/s13075-017-1507-6 (PMC5791187; doi:10.1186/s13075-017-1507-6)
Supplement: Supplementary file 4 — Supplementary tables for comparing the baseline features between different groups. (PDF 298 kb) [file 13075_2017_1507_MOESM4_ESM.pdf]

Supple Table 1 Differences in baseline features between clinically relapsing and clinically stable patients

	Clinically relapsing patients (n = 6)	Clinically stable patients (n = 22)	P
Number of involved SGs	6.00 ± 0.00	5.05 ± 1.49	0.141
Extra-salivary involvement, n (%)	6 (100.0)	21 (95.5)	0.595
Log_2 Serum IgG4 level (mg/L)	13.04 ± 1.66	13.18 ± 1.05	0.849
Volume of SMG (cm^3)	15.21 ± 4.97	11.46 ± 4.70	0.236
CT value of SMG (HU)	36.01 ± 4.59	34.02 ± 6.65	0.656
Volume of PG (cm^3)	39.76 ± 14.19	32.57 ± 7.42	0.336
CT value of PG (HU)	4.74 ±17.09	-8.17 ± 16.74	0.112

Supple Table 2 Differences in baseline features between serologically stable and serologically unstable patients

	Serologically unstable patients (*n* = 7)	Serologically stable patients (*n* = 15)	*P*
Number of involved SGs	5.14 ± 1.57	5.00 ± 1.35	0.630
Extra-salivary involvement, *n* (%)	7 (100.0)	14 (93.3)	0.484
Log_2 Serum IgG4 level (mg/L)	13.29 ± 0.81	13.12 ± 1.12	0.680
Volume of SMG (cm^3)	10.44 ± 7.40	11.93 ± 2.97	0.837
CT value of SMG (HU)	39.08 ± 2.50	32.33 ± 6.79	0.053
Volume of PG (cm^3)	36.59 ± 6.53	30.69 ± 7.24	0.091
CT value of PG (HU)	-5.67 ± 14.51	-9.34 ± 18.04	0.680
